# Supplementary material for: Association of gout with brain reserve and vulnerability to neurodegenerative disease
Source: Nat Commun. 2023 May 18;14:2844. doi: 10.1038/s41467-023-38602-6 (PMC10195870; doi:10.1038/s41467-023-38602-6)
Supplement: Supplementary file 3 — Description of Additional Supplementary Files [file 41467_2023_38602_MOESM3_ESM.pdf]

Description of Additional Supplementary Files

Supplementary Data 1: Baseline characteristics of imaging sample by serum urate quintile

Supplementary Data 2: Associations between serum urate and image-derived phenotypes (IDPs) amongst highest two groups of household income

Supplementary Data 3: Time-dependent coefficients for gout on all cause dementia incidence accounting for the competing risk of death

Supplementary Data 4: Instrumental variables for gout and urate.

Supplementary Data 5: Robust Mendelian randomization estimates
