# Supplementary material for: Association of gout with brain reserve and vulnerability to neurodegenerative disease
Source: Nat Commun. 2023 May 18;14:2844. doi: 10.1038/s41467-023-38602-6 (PMC10195870; doi:10.1038/s41467-023-38602-6)
Supplement: Supplementary file 9 — Reporting Summary [file 41467_2023_38602_MOESM9_ESM.pdf]

nature portfolio

Corresponding author(s): Anya Topiwala

Last updated by author(s): 4/26/2023

Reporting Summary

Nature Portfolio wishes to improve the reproducibility of the work that we publish. This form provides structure for consistency and transparency in reporting. For further information on Nature Portfolio policies, see our Editorial Policies and the Editorial Policy Checklist.

Statistics

For all statistical analyses, confirm that the following items are present in the figure legend, table legend, main text, or Methods section.

n/a	Confirmed	
☐	☒	The exact sample size (*n*) for each experimental group/condition, given as a discrete number and unit of measurement
☐	☒	A statement on whether measurements were taken from distinct samples or whether the same sample was measured repeatedly
☐	☒	The statistical test(s) used AND whether they are one- or two-sided<br>*Only common tests should be described solely by name; describe more complex techniques in the Methods section.*
☐	☒	A description of all covariates tested
☐	☒	A description of any assumptions or corrections, such as tests of normality and adjustment for multiple comparisons
☐	☒	A full description of the statistical parameters including central tendency (e.g. means) or other basic estimates (e.g. regression coefficient) AND variation (e.g. standard deviation) or associated estimates of uncertainty (e.g. confidence intervals)
☐	☒	For null hypothesis testing, the test statistic (e.g. *F*, *t*, *r*) with confidence intervals, effect sizes, degrees of freedom and *P* value noted<br>*Give P values as exact values whenever suitable.*
☒	☐	For Bayesian analysis, information on the choice of priors and Markov chain Monte Carlo settings
☒	☐	For hierarchical and complex designs, identification of the appropriate level for tests and full reporting of outcomes
☐	☒	Estimates of effect sizes (e.g. Cohen's *d*, Pearson's *r*), indicating how they were calculated

Our web collection on statistics for biologists contains articles on many of the points above.

Software and code

Policy information about availability of computer code

Data collection	No software used
Data analysis	R version 4.1.2 was used for most analysis (standard packages - survival v3.5-5, cmprsk v2.2-11, MendelianRandomization v0.7.0, TwoSampleMR v0.4.26, forester 0.5.0, ggforestplot available here: https://github.com/NightingaleHealth/ggforestplot). For VBM analyses, BLM was used (details in SMethods). Code for the VBM analyses performed is available here: https://github.com/TomMaullin/BLMM.

For manuscripts utilizing custom algorithms or software that are central to the research but not yet described in published literature, software must be made available to editors and reviewers. We strongly encourage code deposition in a community repository (e.g. GitHub). See the Nature Portfolio guidelines for submitting code & software for further information.

Data

Policy information about availability of data

All manuscripts must include a data availability statement. This statement should provide the following information, where applicable:

- Accession codes, unique identifiers, or web links for publicly available datasets
- A description of any restrictions on data availability
- For clinical datasets or third party data, please ensure that the statement adheres to our policy

Full pseudonymized participant data cannot be openly shared under the material transfer agreement with UK Biobank and ethics approval. Other researchers can apply

for UK Biobank data to answer specific research questions. Further information about applying for data access can be obtained from the UK Biobank website (https://www.ukbiobank.ac.uk) or by emailing UK Biobank (ukbiobank@ukbiobank.ac.uk). Genetic summary statistics for serum urate, gout and brain imaging measures are freely available. Source data for figures are provided with this paper.

Human research participants

Policy information about studies involving human research participants and Sex and Gender in Research.

Reporting on sex and gender	Participants' sex (on basis of self-report) was used for descriptive purposes and as a covariate in analyses. Sex-separate analyses was not been performed due to the few numbers of females with gout/hyperuricaemia. In response to reviewer request sex-stratified MRI analyses were performed.
Population characteristics	Middle-aged male and female adults in UKB. Those with gout diagnoses (identified by ICD10 diagnosis in linked records) were compared to those without. Full details of characteristics are given in STable 2.
Recruitment	UK Biobank recruited middle-aged adults who responded to an invitation. As such there is a healthy volunteer bias, as described in the study limitations. This bias may mean the results are not generalisation to other populations.
Ethics oversight	UK Biobank has approval from the North West Multi-centre Research Ethics Committee (MREC) as a Research Tissue Bank (RTB) approval.

Note that full information on the approval of the study protocol must also be provided in the manuscript.

Field-specific reporting

Please select the one below that is the best fit for your research. If you are not sure, read the appropriate sections before making your selection.

☒ Life sciences ☐ Behavioural & social sciences ☐ Ecological, evolutionary & environmental sciences

For a reference copy of the document with all sections, see nature.com/documents/nr-reporting-summary-flat.pdf

Life sciences study design

All studies must disclose on these points even when the disclosure is negative.

Sample size	Sample size was determined for observational analyses by number of participants with complete data. Size of MR analyses was determined by largest available GWAS sizes.
Data exclusions	Prevalent dementia cases (to reduce risk of reverse causation).
Replication	Observational and MR analyses were triangulated for better causal inference. No direct replication as no comparable sample to UKB.
Randomization	Participants were not randomized in observational analyses, where all known and available covariates were controlled for in the regression models. In the genetic analyses, MR, is a pseudo-experimental approach which exploits the natural randomization of alleles at conception.
Blinding	Data collection was performed by the UKB study, who were naive to this study focus, independently of authors undertaking the analyses. The investigator who performed the analysis and wrote the manuscript was not blinded to the group.

Reporting for specific materials, systems and methods

We require information from authors about some types of materials, experimental systems and methods used in many studies. Here, indicate whether each material, system or method listed is relevant to your study. If you are not sure if a list item applies to your research, read the appropriate section before selecting a response.

Materials & experimental systems

n/a	Involved in the study
☒	☐ Antibodies
☒	☐ Eukaryotic cell lines
☒	☐ Palaeontology and archaeology
☒	☐ Animals and other organisms
☒	☐ Clinical data
☒	☐ Dual use research of concern

Methods

n/a	Involved in the study
☒	☐ ChIP-seq
☒	☐ Flow cytometry
☐	☒ MRI-based neuroimaging

Magnetic resonance imaging

Experimental design

Design type	Structural, diffusion, swMRI only
Design specifications	N/A
Behavioral performance measures	N/A

Acquisition

Imaging type(s)	Structural, diffusion, swMRI
Field strength	3T
Sequence & imaging parameters	UKB protocol detailed in Miller et al. 2016. e.g. T1: FOV 256mm, 1x1x1mm, TR=2000ms; dMRI 2x2x2mm; swMRI 0.8x0.8x3mm, T=27ms.
Area of acquisition	All IDPs and whole brain for VBM

Diffusion MRI ☒ Used ☐ Not used

Parameters: 50 directions, multiband

Preprocessing

Preprocessing software	Detailed in UKB prptocol paper. Primarily FSL tools. For structural: removal of face, brain extraction.
Normalization	Linear alignment to MNI152 template, nonlinear warping to template.
Normalization template	Standard MNI152 brain template
Noise and artifact removal	Extensive quality control pipeline as specified in UKB protocol, including measures of signal-to-noise ratio in different modalities to automatically identify problematic data.
Volume censoring	Automated machine learning system used to flag problematic data on basis of IDPs/quality measures.

Statistical modeling & inference

Model type and settings	Mass univariate
Effect(s) tested	Multiple regression

Specify type of analysis: ☐ Whole brain ☐ ROI-based ☒ Both

Anatomical location(s): All IDPs

Statistic type for inference (See Eklund et al. 2016)	Voxel-wise
Correction	FDR and Bonferroni (for IDPs)

Models & analysis

n/a	Involved in the study
☒	☐ Functional and/or effective connectivity
☒	☐ Graph analysis
☒	☐ Multivariate modeling or predictive analysis
